# Adherence to Burn Center Referral Criteria for Pediatric Burns

**DOI:** 10.1001/jamanetworkopen.2025.59159

**Published:** 2026-02-12

**Authors:** Eduardo Gus, Teresa To, Joel Fish, Christina Diong, Natasha Saunders

**Affiliations:** 1 Division of Plastic and Reconstructive Surgery, The Hospital for Sick Children, Toronto, Canada; 2 Department of Surgery, Temerty Faculty of Medicine, University of Toronto, Toronto, Canada; 3 Edwin S.H. Leong Centre for Healthy Children, University of Toronto, Toronto, Canada; 4 Child Health Evaluative Sciences, SickKids Research Institute, Toronto, Canada; 5 Institute of Health Policy, Management and Evaluation, Dalla Lana School of Public Health, University of Toronto, Toronto, Canada; 6 ICES, Toronto, Canada; 7 Department of Pediatrics, Temerty Faculty of Medicine, University of Toronto, Toronto, Canada

## Abstract

**Importance:**

Burn injuries are a leading cause of emergency department visits and hospitalizations among children and adolescents. Referral to specialized burn centers is recommended for severe injuries, but adherence to referral guidelines in pediatric care remains uncertain.

**Objective:**

To evaluate the association between pediatric burn characteristics, including referral criteria, and the setting of care, burn center vs non–burn center.

**Design, Setting, and Participants:**

This population-based cohort study used linked health and administrative databases among all children and adolescents aged 0 to 17 years with a hospital visit for burn injury residing in Ontario, Canada (population, approximately 16 million), from April 1, 2003, to March 31, 2023. Data were analyzed from November 2023 to September 2024.

**Exposure:**

Presence and number of 1 or more of 6 measurable burn center referral criteria: burns over more than 10% of the total body surface area, full-thickness burns, critical anatomical areas, and inhalation, chemical, or electrical injuries.

**Main Outcome and Measure:**

Receipt of care at a specialized burn center vs a non–burn center. Adjusted rate ratios (aRRs) were estimated using modified Poisson regression.

**Results:**

Among 79 782 youths with burn injuries (median [IQR] age, 4 [1-12] years; 44 191 male [55.4%]), 16 164 individuals (20.3%) were rural residents, 19 067 individuals (23.9%) lived in the lowest material resource quintile, and 1636 individuals (2.1%) were nonrefugee immigrants. There were 34 812 youths (43.6%) who met at least 1 referral criterion, but only 7533 of these (21.6%) were treated at a burn center. Youths meeting any referral criterion were more likely to be treated at a burn center compared with those who met no referral criteria (aRR, 1.50; 95% CI, 1.46-1.54). The likelihood of burn center treatment increased with the number of criteria (1 criterion: aRR, 1.27; 95% CI, 1.23-1.30; 2 criteria: aRR, 2.63; 95% CI, 2.51-2.75; ≥3 criteria: aRR, 4.71; 95% CI, 4.32-5.15). Inhalation (aRR, 1.88; 95% CI, 1.21-2.91) and chemical (aRR, 1.80; 95% CI, 1.65-1.97) injuries had the largest aRRs in the association with burn center care.

**Conclusions and Relevance:**

In this study, burn center referral criteria were associated with increased likelihood of specialized care, although the application of criteria was inconsistent. These findings suggest that enhancing adherence to referral guidelines and addressing geographic and systemic barriers are essential to improving equitable access to specialized pediatric burn care.

## Key Points

**Question:**

Is the presence of pediatric burn center referral criteria associated with treatment at specialized burn centers in Ontario, Canada?

**Findings:**

In this cohort study of 79 782 children and adolescents with burn injuries, 43.5% met at least 1 burn center referral criterion; however, only 21.6% of these individuals received specialized care. Youths meeting 1 or more referral criteria were significantly more likely to be treated at a burn center.

**Meaning:**

In this study, adherence to burn center referral criteria in Ontario was low, suggesting missed opportunities for specialized care and a need for improved referral practices.

## Teaser

This cohort study investigates the association between pediatric burn characteristics, including referral criteria, and receipt of care at a specialized burn center.

## Introduction

Burns injuries are a leading cause of emergency department visits and hospitalizations among children and adolescents in Ontario, Canada.^1,2,3^ These injuries range in severity from minor, superficial thermal burns, often managed at home or in primary care settings, to more extensive or deep injuries that necessitate acute hospital-based care. In cases of severe burns and for those that involve functionally critical areas or are caused by nonthermal mechanisms, referral to specialized burn centers is recommended.^4,5^

Specialized burn centers are designed to deliver high-intensity, multidisciplinary care tailored to the complex needs of patients with severe burns.^6^ These centers typically are located within tertiary care hospitals, have a high volume of patients with burns, and are staffed by teams that include surgeons, nurses, therapists, social workers, dieticians, child life specialists, and researchers. This structure enables a coordinated, evidenced-based approach that extends from prehospital stabilization through long-term rehabilitation and social reintegration.^6,7^ Such specialized and high-volume settings for burn and trauma care have been shown to be associated with better outcomes.^8,9,10,11,12^

Referral to a specialized burn center is guided by established clinical criteria; although these are periodically updated by regional health authorities, they consistently reflect the extent of injury or potential for functional impairment and the need for specialized resources.^4,5,13,14^ In Ontario, these clinical criteria include burns on more than 10% of the total body surface area (TBSA), full-thickness burns, burns to critical anatomical regions (eg, face, hands, feet, genitalia, perineum, or major joints), electrical or chemical burns, inhalation injuries, burns complicated by comorbid conditions or trauma, and pediatric cases requiring specialized infrastructure or support services (eg, child abuse concerns or special rehabilitative needs). Ontario burn center consultation guidelines are broadly concordant with contemporary recommendations from the American Burn Association (ABA), European Burn Association, and Australia and New Zealand Burn Association,^4,5,13,14^ with all 4 systems emphasizing referral for larger TBSA burns, full-thickness injuries, burns to critical anatomic regions, inhalation, electrical and chemical injuries, and patients with significant comorbidities or associated trauma. Minor differences relate mainly to how age-specific TBSA thresholds are operationalized, the explicit listing of high-risk comorbidities in Ontario, and the inclusion of nonaccidental burns and pregnancy in Australia and New Zealand Burn Association guidelines rather than to any substantive divergence in which patients should be treated at burn centers.

It is unknown how well clinicians adhere to burn center referral criteria in Ontario. This is important given that aligning burn care delivery to patient needs ensures system efficiency and optimal patient outcomes. The objective of this study was to test the association of patient and clinical characteristics, particularly those outlined in burn center referral criteria, with the treatment setting by comparing characteristics of patients treated at specialized burn center vs non–burn center facilities. Because consultation and referral guidance is not prescriptive and is applied with clinical judgement, *adherence* in this study denotes measurable alignment with coded criteria rather than clinical appropriateness.

## Methods

### Study Design, Population, and Data Sources

We conducted a population-based open cohort study in Ontario, Canada’s most populous province, home to nearly 3 million children and adolescents. Ontario operates under a universal, publicly funded health care system that provides medically necessary services from physicians and hospitals without direct costs to residents. We used data from several health administrative and demographic datasets held at ICES, an independent, not-for-profit organization with the objective of creating evidence to inform policy and health care delivery (eTable 1 in Supplement 1).^15^ The use of these data was authorized under section 45 of the Ontario Personal Health Information Privacy Act, which authorizes ICES to collect personal health information without patient consent for health system planning and evaluation and is exempt from research ethics board review at The Hospital for Sick Children.^16^ This study followed the Reporting of Studies Conducted Using Observational Routinely-Collected Health Data (RECORD) statement.^17^

We included a cohort of Ontario residents aged 0 to 17 years who had complete linkage with the Ontario provincial health care registry and sustained a burn injury from April 1, 2003, to March 31, 2023. We ascertained burn injuries using validated *International Statistical Classification of Diseases and Related Health Problems, Tenth Revision, With Canadian Modification* (*ICD-10-CA*) discharge diagnosis codes for burn injuries recorded in emergency department and hospital discharge records (eTable 2 in Supplement 1).^18,19,20^ We excluded individuals with burn injuries with no linkage to an institution number. We also excluded recurrent burn injuries to ensure that only incident burns were measured. For population denominators, we identified youths living in Ontario eligible for provincial health insurance in the Ontario health system registry on April 1 of each study year.

### Measures

Our main exposure was the presence of 1 or more of 6 identifiable burn center referral criteria using health administrative data. These included partial-thickness burns involving more than 10% TBSA, full-thickness burns, burns involving critical anatomical areas (ie, face, hands, feet, perineum or genitalia, or major joints), inhalation injuries, chemical injuries, and electrical injuries (eTable 2 in Supplement 1). Our primary outcome measure was the setting of definitive burn care operationalized as a burn center vs a non–burn center. Pediatric and adult burn centers were considered given that adult facilities often care for adolescents. We classified patients as having received care at a burn center if they had at least 1 encounter at 1 of 6 designated burn care institutions in Ontario within 6 weeks of the first health care encounter for a burn injury. Of these institutions, 3 are stand-alone pediatric centers. This definition reflects that injuries that are superficial and heal spontaneously usually do so in 2 to 3 weeks and those that do not heal or are deep and require surgical treatment will continue to be managed in a hospital setting (outpatient, day-surgery, or hospitalization settings) for approximately 2 to 6 weeks. A timeline of up to 6 weeks between the first burn encounter and a health care encounter at a burn center would typically capture appropriate and late referrals. Covariates included demographic characteristics (age and sex), indicators of social vulnerability (neighborhood material resource quintile, maternal age at childbirth, and immigrant status), care accessibility factors (urban vs rural residence and regional availability of a burn center), and year of burn injury (eTable 2 in Supplement 1).

### Statistical Analysis

We summarized cohort baseline characteristics using descriptive statistics, including frequencies, proportions, and population rates (eTable 3 in Supplement 1). We computed standardized differences to compare characteristics of youths treated at burn centers and non–burn centers, with differences greater than 0.10 considered meaningful.^21^

We used modified Poisson regression to estimate adjusted rate ratios (aRRs) and 95% CIs for the likelihood of receiving treatment at a burn center for injuries meeting burn center referral criteria. We constructed 3 sequential models with increasing levels of adjustment. Model 1 adjusted for time; model 2 adjusted for time and sociodemographic characteristics; model 3 adjusted for time, sociodemographic characteristics, social vulnerability characteristics, and care accessibility factors.

To account for the presence of multiple referral criteria in any given burn injury, additional analyses modified our exposure definition to include the number of burn center referral criteria (no criterion [referent], 1 criterion, 2 criteria, and ≥3 criteria). To estimate the risk of burn center treatment associated with each individual criterion, we modified our exposure definition to examine the association between each mutually exclusive burn center referral criterion and burn center treatment. Where more than a single criterion was present, we considered this group separately as an exposure group in models.

All analyses used SAS statistical software version 9.4 (SAS Institute). Data were analyzed from November 2023 to September 2024. We defined statistical significance with an α of .05 or if 95% CIs did not cross 1.0.

## Results

### Cohort Sociodemographic and Clinical Characteristics

After exclusions, there were 79 782 youths with burn injuries (median [IQR] age, 4 [1-12] years; 44 191 male [55.4%]; 136 burn injuries per 100 000 population) (Table 1; eFigure in Supplement 1), among whom 16 164 youths (20.3%) were from rural areas and 19 067 youths (23.9%) lived in neighborhoods in the most material resource–deprived quintile, and 1636 individuals (2.1%) were nonrefugee immigrants (Table 1). There were 34 812 burn injuries (43.6%) that had at least 1 burn center referral criterion, the most common of which were burns involving critical anatomical areas (27 546 injuries [34.5%]), full-thickness burns (5308 injuries [6.7%]), and chemical injuries (2636 injuries [3.3%]).

**Table 1.** Baseline Burn Injury and Patient Characteristics

Characteristic	Patients, No. (%)	Rate per 100 000 population
Overall burn injuries	79 782 (100)	136
Characteristics of burn injuries		
Burn center referral criteria, No.		
None	44 970 (56.4)	77
Any	34 812 (43.6)	59
1	29 768 (37.3)	51
2	4459 (5.6)	7.6
≥3	585 (0.7)	1.0
Individual burn center referral criteria (not mutually exclusive)^a^		
No burn center referral criterion	44 970 (56.4)	77
Partial-thickness burns >10% TBSA	2474 (3.1)	4.2
Full-thickness burns	5308 (6.7)	9.1
Burns to special anatomic areas	27 546 (34.5)	47
Inhalation injury	85 (0.1)	0.1
Chemical injury	2636 (3.3)	4.5
Electrical injury	2419 (3.0)	4.1
≥2 Burn center referral criteria	5044 (6.3)	8.6
Patient characteristics		
Age, y		
Median (IQR)	4 (1-12)	NA
0 to <1	8015 (10.0)	144
1 to 4	32 853 (41.2)	286
5 to 12	18 143 (22.7)	73
13 to 17	20 771 (26.0)	125
Sex		
Male	44 191 (55.3)	147
Female	35 591 (44.7)	125
Material resources, quintile		
1 (Least deprived)	13 483 (16.9)	111
2	14 933 (18.7)	124
3	15 260 (19.1)	137
4	15 498 (19.4)	148
5 (Most deprived)	19 067 (23.9)	159
Missing	1541 (1.9)	214
Youths of adolescent mothers (≤19 y)		
Yes	3845 (4.8)	236
No	60 712 (76.1)	142
Missing	15 225 (19.1)	108
Residence type		
Rural	16 164 (20.3)	258
Urban	63 513 (79.6)	122
Missing	105 (0.1)	142
Health region with burn center		
Yes	30 000 (37.6)	149
No	49 782 (62.4)	129
Immigration status		
Nonimmigrant	77 444 (97.1)	142
Nonrefugee immigrant	1636 (2.1)	50
Refugee immigrant	702 (0.9)	93

Abbreviations: NA, not applicable; TBSA, total body surface area.

^a^ Mutually exclusive values were 44 970 patients (56.4%) for no burn center referral criteria, 1097 patients (1.4%) for partial-thickness burns more than 10% TBSA, 2286 patients (2.9%) for full-thickness burns, 23 030 patients (28.9%) for burns to special anatomic areas, 56 patients (0.1%) for inhalation injury, 1192 patients (1.5%) for chemical injury, 2107 patients (2.6%) for electrical injury, and 5044 patients (6.3%) for 2 or more burn center referral criteria.

### Treatment at Burn Centers and Non–Burn Centers

Of all pediatric patients with burns, 13 531 individuals (17.0%) were treated at burn centers. Of all youths treated at burn centers, 7533 individuals (55.6%) had burn center referral criteria. In contrast, of the remaining 66 251 youths with burns (83.0%) treated at non–burn centers, 27 279 individuals (41.2%) had burn center referral criteria (Table 2). Burn centers treated a greater proportion of infants and preschool-age children, urban-dwelling youths, and those living in health regions with burn centers compared with non–burn centers.

**Table 2.** Characteristics of Pediatric Burn Injuries Treated at Burn Centers vs Non–Burn Centers

Characteristic	Burn injuries, No. (%) (N = 79 782)		SDiff
	Treated at burn centers (n = 13 531)	Treated at non–burn centers (n = 66 251)	
Characteristics of burn injuries			
Burn center referral criteria, No.			
None	5998 (44.3)	38 972 (58.8)	0.29
Any	7533 (55.7)	27 279 (41.2)	0.29
1	5398 (39.9)	24 370 (36.8)	0.06
2	1748 (12.9)	2711 (4.1)	0.32
≥3	387 (2.9)	198 (0.3)	0.21
Individual burn center referral criteria (not mutually exclusive)			
None	5998 (44)	38 972 (59)	0.29
Partial-thickness burns >10% TBSA	1063 (7.9)	1411 (2.1)	0.27
Full-thickness burns	1890 (14.0)	3418 (5.2)	0.30
Burns to special anatomic areas	5976 (44.2)	21 570 (32.6)	0.24
Inhalation injury	38 (0.3)	47 (0.1)	0.05
Chemical injury	795 (5.9)	1841 (2.8)	0.15
Electrical injury	317 (2.3)	2102 (3.2)	0.05
Patient characteristics			
Age, y			
Median (IQR)	2 (0-8)	4 (1-13)	0.39
0 to <1	2057 (15.2)	5958 (9.0)	0.19
1 to 4	6675 (49.3)	26 178 (39.5)	0.20
5 to 12	2830 (20.9)	15 313 (23.1)	0.05
13 to 17	1969 (14.6)	18 802 (28.4)	0.34
Sex			
Male	7644 (56.5)	36 547 (55.2)	0.03
Female	5887 (43.5)	29 704 (44.8)	0.03
Material resources, quintile			
1 (Least deprived)	2920 (21.6)	10 563 (15.9)	0.14
2	2249 (16.6)	12 684 (19.1)	0.07
3	2077 (15.3)	13 183 (19.9)	0.12
4	2341 (17.3)	13 157 (19.9)	0.07
5 (Most deprived)	3765 (27.8)	15 302 (23.1)	0.11
Missing	179 (1.3)	1362 (2.1)	0.06
Youths of adolescent mothers (≤19 y old)			
Yes	533 (3.9)	3312 (5.0)	0.05
No	10 660 (78.8)	50 052 (75.5)	0.08
Missing	2338 (17.3)	12 887 (19.5)	0.06
Residence type			
Rural	765 (5.7)	15 399 (23.2)	0.52
Urban	12 725 (94.0)	50 788 (76.7)	0.51
Missing	41 (0.3)	64 (0.1)	0.05
Health region with burn center			
Yes	9754 (72.1)	20 246 (30.6)	0.91
No	3777 (27.9)	46 005 (69.4)	0.91
Immigration status			
Nonimmigrant	13 064 (96.5)	64 380 (97.2)	0.04
Nonrefugee immigrant	257 (1.9)	1379 (2.1)	0.01
Refugee immigrant	210 (1.6)	492 (0.7)	0.08

Abbreviations: SDiff, standardized difference; TBSA, total body surface area.

### Main Analysis

Among 44 970 youths with burns that did not meet any burn center referral criteria, 5998 individuals (13.3%) were treated at a burn center compared with 7533 of 34 812 youths (21.6%) who met at least 1 referral criterion (aRR, 1.50; 95% CI, 1.46-1.54) (Table 3; eTable 4 in Supplement 1). With increasing numbers of burn center referral criteria, the likelihood of treatment at a burn center increased (single criterion: 5398 of 29 768 youths [18.1%]; aRR, 1.27; 95% CI, 1.23-1.30; 2 criteria: 1748 of 4459 youths [39.2%]; aRR, 2.63; 95% CI, 2.51-2.75; ≥3 criteria: 387 of 585 youths [66.2%]; aRR, 4.71; 95% CI, 4.32-5.15) compared with no burn center referral criteria met (5998 of 44 970 youths [13.3%]) (Table 4; eTable 5 in Supplement 1).

**Table 3.** Likelihood of Being Treated at a Burn Center With or Without Burn Center Referral Criteria

Burn center referral criteria	Burn injuries		aRR (95% CI)^a^		
	Total, No. (N = 79 782)	Treated at burn centers, No. (%) (n = 13 531 [17.0%])	Model 1^b^	Model 2^c^	Model 3^d^
None	44 970	5998 (13.3)	1 [Reference]	1 [Reference]	1 [Reference]
Any	34 812	7533 (21.6)	1.61 (1.56-1.66)	1.53 (1.48-1.58)	1.50 (1.46-1.54)

Abbreviation: aRR, adjusted rate ratio.

^a^ The full model with parameter estimates for covariates is in eTable 4 in Supplement 1.

^b^ Model 1 adjusted for time (fiscal year).

^c^ Model 2 adjusted for time (fiscal year) and demographics (age and sex).

^d^ Model 3 adjusted for time (fiscal year), demographics (age and sex), and social vulnerability and accessibility factors (material resource quintiles, youths of adolescent mothers, rural residence, health region with or without burn centers, and immigration status).

**Table 4.** Likelihood of Being Treated at a Burn Center With ≥1 Burn Center Referral Criteria^a^

Burn center referral criteria, No.	Burn injuries		aRR (95% CI)		
	Total, No. (N = 79 782)	Treated at burn centers, No. (%) (n = 13 531 [17.0%])	Model 1^b^	Model 2^c^	Model 3^d^
None	44 970	5998 (13.3)	1 [Reference]	1 [Reference]	1 [Reference]
1	29 768	5398 (18.1)	1.36 (1.31-1.40)	1.30 (1.25-1.34)	1.27 (1.23-1.30)
2	4459	1748 (39.2)	2.83 (2.71-2.95)	2.62 (2.51-2.73)	2.63 (2.51-2.75)
≥3	585	387 (66.2)	4.72 (4.44-5.03)	4.40 (4.12-4.70)	4.71 (4.32-5.15)

Abbreviation: aRR, adjusted rate ratio.

^a^ The full model with parameter estimates for covariates is in eTable 5 in Supplement 1.

^b^ Model 1 adjusted for time (fiscal year).

^c^ Model 2 adjusted for time (fiscal year) and demographics (age and sex).

^d^ Model 3 adjusted for time (fiscal year), demographics (age and sex), and social vulnerability and accessibility factors (material resource quintiles, youths of adolescent mothers, rural residence, health region with or without burn centers, and immigration status).

### Burn Center Referral Criteria–Specific Analysis

Compared with 5998 of 44 970 youths (13.3%) who did not meet any referral criteria and were treated at a burn center (reference group), youths with most individual, mutually exclusive burn center referral criteria were more likely to receive care at a burn center. Partial-thickness burns involving more than 10% TBSA accounted for 1097 of these burns, of which 247 burns (22.5%) were treated at burn centers (aRR, 1.66; 95% CI, 1.49-1.84). Full-thickness burns showed a similar treatment rate (555 of 2286 youths [24.3%]; aRR, 1.66; 95% CI, 1.54-1.78). Burns involving critical anatomical areas were associated with a moderate increase in burn center treatment (4012 of 23 030 youths [17.4%]; aRR, 1.21; 95% CI, 1.17-1.25). Although inhalation injuries were rare, they had a relatively high burn center treatment rate (14 of 56 youths [25.0%]; aRR, 1.88; 95% CI, 1.21-2.91). Chemical injuries (318 of 1192 youths [26.7%]; aRR, 1.80; 95% CI, 1.65-1.97) were also associated with burn center treatment. In contrast, youths with electrical injuries were not more likely to be treated at a burn center than the reference group. Youths who met 2 or more of these referral criteria had the highest likelihood of burn center treatment, with 2135 of 5044 individuals (42.3%) receiving specialized care and an aRR of 2.86 (95% CI, 2.75-2.98) compared with patients who met no criteria (Table 5; eTable 6 in Supplement 1).

**Table 5.** Likelihood of Being Treated at a Burn Center With Mutually Exclusive Specific Burn Center Referral Criteria^a^

Burn center referral criteria (mutually exclusive)	Burn injuries		aRR (95% CI)		
	Total, No. (N = 79 782)	Treated at burn centers, No. (%) (n = 13 531 [17.0%])	Model 1^b^	Model 2^c^	Model 3^d^
None	44 970	5998 (13.3)	1 [Reference]	1 [Reference]	1 [Reference]
>10% TBSA partial-thickness burn only	1097	247 (22.5)	1.65 (1.48-1.85)	1.52 (1.36-1.70)	1.66 (1.49-1.84)
Full-thickness burn only	2286	555 (24.3)	1.72 (1.59-1.85)	1.68 (1.56-1.81)	1.66 (1.54-1.78)
Burns to special anatomic areas only	23 030	4012 (17.4)	1.32 (1.27-1.37)	1.25 (1.20-1.29)	1.21 (1.17-1.25)
Inhalation injury only	56	14 (25.0)	1.86 (1.20-2.89)	2.15 (1.40-3.29)	1.88 (1.21-2.91)
Chemical injury only	1192	318 (26.7)	1.93 (1.75-2.12)	1.83 (1.67-2.01)	1.80 (1.65-1.97)
Electrical injury only	2107	252 (12.0)	0.88 (0.78-0.99)	0.91 (0.81-1.02)	0.93 (0.84-1.04)
≥2 Criteria	5044	2135 (42.3)	3.05 (2.93-3.18)	2.83 (2.72-2.95)	2.86 (2.75-2.98)

Abbreviations: aRR, adjusted rate ratio; TBSA, total body surface area.

^a^ The full model with parameter estimates for covariates is in eTable 6 in Supplement 1.

^b^ Model 1 adjusted for time (fiscal year).

^c^ Model 2 adjusted for time (fiscal year) and demographics (age and sex).

^d^ Model 3 adjusted for time (fiscal year), demographics (age and sex), and social vulnerability and accessibility factors (material resource quintiles, youths of adolescent mothers, rural residence, health region with or without burn centers, and immigration status).

## Discussion

In this large population-based cohort study of nearly 80 000 pediatric burn injuries, we found that a substantial proportion of youths met established burn center referral criteria but most individuals with such criteria were treated at non–burn centers. While the presence of burn center referral criteria was associated with an increased likelihood of treatment at a burn center, alignment of practice to these criteria was generally poor. Almost half of youths with burn injuries met at least 1 burn center referral criterion, with burns involving critical anatomical areas being the most common. However, only 55.6% of youths treated at burn centers met any referral criteria, while 41.2% of those treated at non–burn centers also met such criteria. These findings suggest both underuse and overuse of specialized burn care, despite the existence of standardized referral guidelines. The mismatch between injury severity and treatment setting may be associated with negative outcomes in both directions. Overuse of burn centers may be associated with unnecessary use of specialized resources for cases that could be managed effectively in general health care settings. Conversely, underuse means some patients may not receive the level of care their injuries require, with an associated increase in the risk of complications. Both scenarios are harmful, not only to patient outcomes but also to the health care system as a whole.

Youths with increasing numbers of referral criteria were progressively more likely to receive care at burn centers, supporting the clinical relevance of the referral guidelines and clinician recognition of the cumulative severity or complexity of injuries in guiding referral decisions. However, even among youths meeting 3 or more criteria, one-third were not treated at a burn center. This highlights potential gaps in the triage and referral of complex burn injuries. Our criteria-specific analysis revealed that some injuries, such as inhalation or chemical burns, were associated with large increases in the likelihood of burn center care; others, such as electrical injuries and burns involving critical areas, were not, despite their inclusion in referral guidelines. This may reflect variation in injury severity, misclassification, or inconsistent application of criteria.

Most pediatric burn injuries in our cohort, including many that met referral criteria, were managed at nonspecialized facilities. Similarly low adherence has been reported elsewhere. A 2016 US study^22^ found that 8.2% of pediatric burns meeting ABA criteria were transferred to burn centers, with most patients receiving definitive care at local hospitals. However, differences in health care financing limit direct comparisons. Studies from Canada and the United Kingdom also report low adherence, although methods vary, with most based on single-center or adult data, limiting their ability to be benchmarked with population-level data.^23,24,25,26^ Moreover, given the predominance of outpatient treatment in pediatric burns, studies focused solely on hospitalized cases may underestimate the scope of underreferral.^27,28,29,30^

Sociodemographic disparities were also associated with access to specialized care. Infants and toddlers had the highest likelihood of being treated at burn centers, while adolescents were more often treated at nonspecialized facilities. Similar patterns have been observed in the US, likely reflecting factors such as clinician comfort, availability of pediatric-specific resources (eg, child life services and pediatric anesthesia), and clinical expertise in managing injuries in growing youths.^22,31^ Youths living in urban areas and regions with a burn center were more likely to receive specialized care. This aligns with previous reports from Canada^32^ and may reflect reduced travel burden and established referral pathways. All burn centers in Ontario are located in the south (Toronto, Ottawa, Hamilton, and London), leaving northern populations several hours from specialized care, even by air transport. Past provincial efforts to regionalize health care may have unintentionally reinforced care boundaries that limit referrals across regions, many of which lack burn centers.^33,34^ Similar findings have been shown in the US, where even modest increases in distance from home to burn center have been associated with substantial financial outcomes, contributing to disparities in access between urban and rural populations.^35^ This raises concerns about geographic and systemic barriers to access, particularly for youths in rural areas or health regions without a burn center. Importantly, youths in our study from the most materially deprived neighborhoods represented a substantial portion of the cohort, with no major differences in the proportion treated at burn centers. This finding may reflect that the single-payer provincial health care system in Ontario may mitigate inequities observed in treatment settings in other jurisdictions.

### Limitations

Despite the strengths of the population-based design that minimizes selection bias and includes 2 decades of data, our study is not without limitations. Some youths who did not meet measurable referral criteria may have had some noncoded indications for burn center care, such as psychosocial needs, comorbidities, complex rehabilitation requirements, poorly controlled pain, or burns associated with trauma. These are not captured in administrative data and likely led to underestimation of referral-eligible cases and overestimation of adherence. Conversely, youths meeting criteria may have been overascertained owing to data limitations (eg, lack of information on low- vs high-voltage electrical injuries or inhalation injuries with vs without cutaneous burns). This underscores the complexity of measuring referral appropriateness and highlights the importance of enhancing data granularity in future studies. While the Ontario consultation criteria did not change during the study period, the ABA criteria were updated in 2022, shifting from strict referral guidelines to consultation guidelines, reflecting the growing role of telehealth in pediatric burn care. As we move forward, it will be important to understand how updated consultation criteria and telehealth have facilitated access to specialty care. Our administrative data did not meaningfully capture the use of telehealth or other remote forms of consultation during the study period, and their use was not commonplace at that time. The impact of these evolutions in the burn referral to consultation criteria and the more widespread adoption of virtual care models are important areas for future research. The focus of this study was on adherence to referral criteria using administrative data, and it did not measure the nuance of how clinical judgment was incorporated in the application of guidelines or decision to transfer a patient to specialized care. Meaningful pediatric outcome measures for burn care are less well established than in adult burn care and, while outside of the scope of this study, remain an important knowledge gap. Further limitations are that residual confounding from unmeasured factors remains possible. Additionally, while burn code validation has been conducted in adults, further validation in pediatric populations is warranted to improve case ascertainment and refine adherence assessments.

## Conclusions

In this cohort study of pediatric burn injuries, burn center referral criteria were generally associated with treatment at specialized centers, but their application in clinical practice was inconsistent. Many eligible youths were treated at nonspecialized centers, highlighting missed opportunities for receipt of care as per guidelines. Improving alignment of clinical practice to referral guidelines and addressing systemic and geographic barriers are essential next steps. Further research should explore clinician- and system-level drivers of referral decisions and evaluate the association of burn center care with outcomes, particularly for youths with multiple qualifying injuries.

## Notes

Supplement 1. eFigure. Flow chart of burn injuries included in the cohort eTable 1. Linked health administrative and demographic datasets eTable 2. Diagnosis codes *International Statistical Classification of Diseases and Related Health Problems, Tenth Revision, With Canadian Modification* (*ICD-10-CA*) for burn injuries eTable 3. Annual population denominators for Ontario on April 1 of fiscal years 2003-2023 eTable 4. Likelihood of being treated at a burn centre for pediatric burn injuries with and without burn center referral criteria in Ontario, Canada, 2003-2023 (with parameters estimates for covariates) eTable 5. Likelihood of being treated at a burn center for pediatric burn injuries with ≥1 burn center referral criteria in Ontario, Canada, 2003-2023 (with parameters estimates for covariates) eTable 6. Likelihood of being treated at a burn center for pediatric burn injuries with mutually exclusive specific burn center referral criteria in Ontario, Canada, 2003-2023 (with parameters estimates for covariates)

Supplement 2.
Data Sharing Statement
