# Supplementary material for: Adherence to Burn Center Referral Criteria for Pediatric Burns
Source: JAMA Netw Open. 2026 Feb 12;9(2):e2559159. doi: 10.1001/jamanetworkopen.2025.59159 (PMC12902878; doi:10.1001/jamanetworkopen.2025.59159)
Supplement: Supplement 1. — eFigure. Flow chart of burn injuries included in the cohort eTable 1. Linked health administrative and demographic datasets eTable 2. Diagnosis codes International Statistical Classification of Diseases and Related Health Problems, Tenth Revision, With Canadian Modification (ICD-10-CA) for burn injuries eTable 3. Annual population denominators for Ontario on April 1 of fiscal years 2003-2023 eTable 4. Likelihood of being treated at a burn centre for pediatric burn injuries with and without burn center referral criteria in Ontario, Canada, 2003-2023 (with parameters estimates for covariates) eTable 5. Likelihood of being treated at a burn center for pediatric burn injuries with ≥1 burn center referral criteria in Ontario, Canada, 2003-2023 (with parameters estimates for covariates) eTable 6. Likelihood of being treated at a burn center for pediatric burn injuries with mutually exclusive specific burn center referral criteria in Ontario, Canada, 2003-2023 (with parameters estimates for covariates) [file jamanetwopen-e2559159-s001.pdf]

Supplemental Online Content

Gus E, To T, Fish J, Diong C, Saunders N. Adherence to burn center referral criteria for pediatric burns. *JAMA Netw Open*. Published online February 11, 2026. doi:10.1001/jamanetworkopen.2025.59159

eFigure. Flow chart of burn injuries included in the cohort
eTable 1. Linked health administrative and demographic datasets
eTable 2. Diagnosis codes *International Statistical Classification of Diseases and Related Health Problems, Tenth Revision, With Canadian Modification* (*ICD-10-CA*) for burn injuries
eTable 3. Annual population denominators for Ontario on April 1 of fiscal years 2003-2023
eTable 4. Likelihood of being treated at a burn centre for pediatric burn injuries with and without burn center referral criteria in Ontario, Canada, 2003-2023 (with parameters estimates for covariates)
eTable 5. Likelihood of being treated at a burn center for pediatric burn injuries with ≥1 burn center referral criteria in Ontario, Canada, 2003-2023 (with parameters estimates for covariates)
eTable 6. Likelihood of being treated at a burn center for pediatric burn injuries with mutually exclusive specific burn center referral criteria in Ontario, Canada, 2003-2023 (with parameters estimates for covariates)

This supplemental material has been provided by the authors to give readers additional information about their work.

© 2026 Gus E et al. *JAMA Network Open*.

eFigure 1. Flow chart of burn injuries included in the cohort

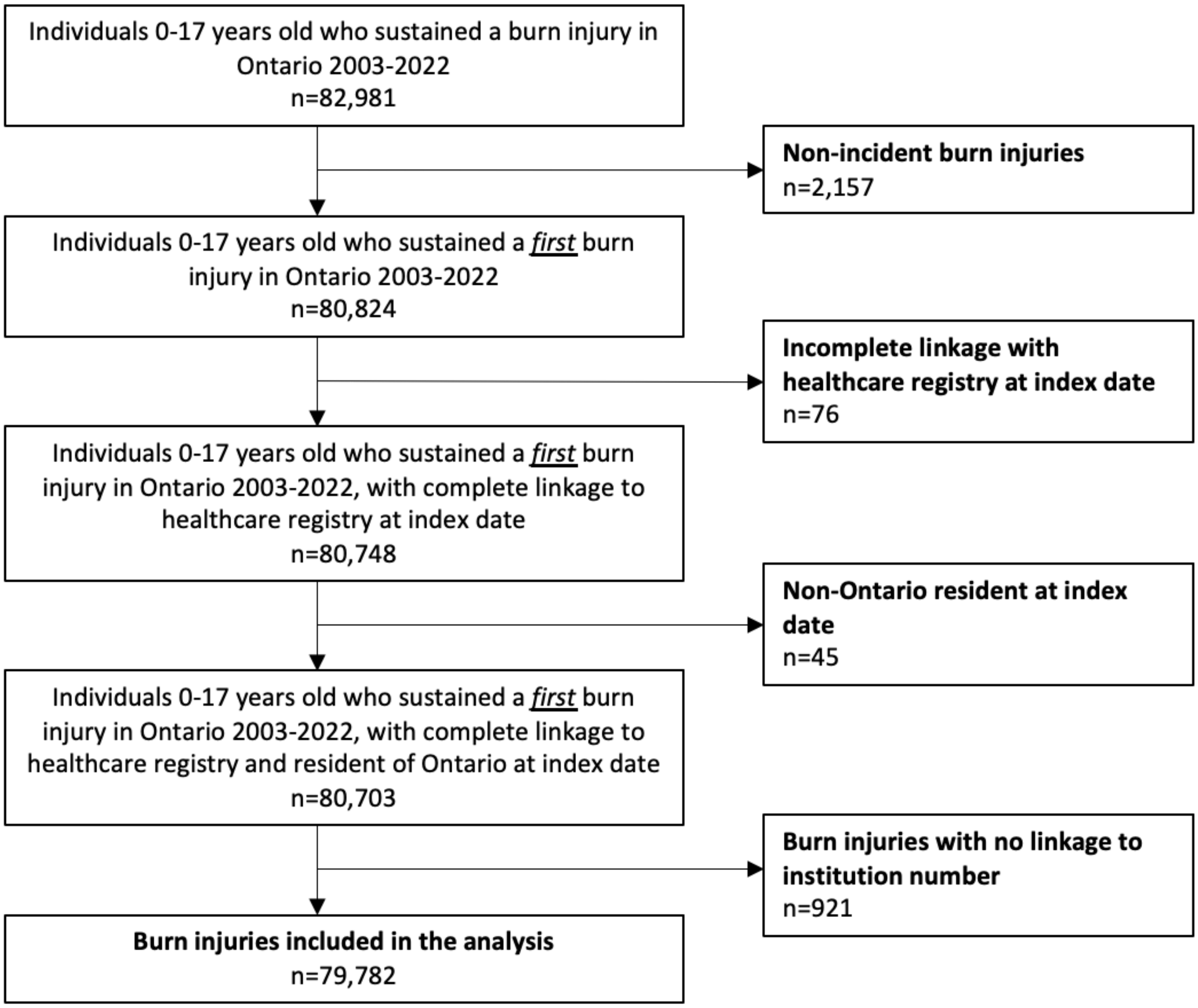

© 2026 Gus E et al. *JAMA Network Open*.

eTable 1. Linked health administrative and demographic datasets

Dataset	Information
**Canadian Institute for Health Information (CIHI) – Discharge Abstract Database (DAD)**	Data from hospital discharges, including deaths, sign-outs and transfers.<br>• Administrative information: institution/hospital number, length of hospital stay, disposition<br>• Clinical Data: diagnoses, interventions, physicians
**Canadian Institute for Health Information (CIHI) - National Ambulatory Care Reporting System (NACRS)**	Data from ambulatory care, same-day surgery (SDS), outpatient clinic and emergency department visits.<br>ED visits and dressing changes under sedation (SDS)
**Registered Persons Database (RPDB)**	Ontario's healthcare registry.<br>• Demographic data: sex, date of birth<br>• Geographic data: municipality, postal code, dissemination area of residence
**Statistics Canada's Canadian Census (CENSUS)**	Information about the population, e.g., location of residence. CENSUS allows for linkage of residential address to geographic-level proxies for socioeconomic status, e.g., material resources from ON-MARG
**Immigration, Refugees, and Citizenship Canada (IRCC) Permanent Residents Database**	Data elements related to immigration in Ontario, e.g., immigration category (non-refugee/refugee immigrants)
**Postal Code^{OM} Conversion File (PCCF)[1]**	The PCCF links postal codes to standard geographic areas (e.g. dissemination areas), allowing for linkage between geographic data elements and neighbourhood socioeconomic characteristics, e.g. material resources from ON-MARG
**Ontario Marginalization Index (ON-MARG)**	Dataset that combines demographic indicators into four distinct dimensions of marginalization: (a) household and dwellings, (b) material resources, (c) age and labour force, and (d) racialized and newcomer populations. We extracted material resources from ON-MARG, which reflects the ability of individuals and communities to access and attain basic material needs, e.g. housing, food, clothing and education
**MOMBABY**	Dataset that links birth mothers to their newborns born in Ontario hospitals through maternal and infant birth hospitalization records. We used MOMBABY to identify children of adolescent mothers (mothers who gave birth under the age of 20 years old)
**Health Care Institutions (INST)**	Dataset that identifies healthcare facilities in Ontario (name, institution number, hospital status, ability to provide urgent care, among others). We used INST to identify the type of institution at which a burn episode was managed (treatment setting).

1 - OM: Postal Code is an official trademark of Canada Post Corporation

© 2026 Gus E et al. *JAMA Network Open*.

eTable 2. Diagnosis codes International Classification of Diseases 10th Revision with Canadian Modifications (ICD-10-CA) for burn injuries

	Variable	Type of variable	Definition
**Main exposure**	Burn centre referral criteria	Categorical and binary	**Definition of burns meeting criteria for treatment at a burn centre: ICD-10-CA discharge diagnosis codes from ED and hospital records**<br><br>**Partial thickness (2nd degree) burn injury > 10% TBSA (total body surface area)**<br>Partial thickness (2nd degree) burn injury by size:<br>T31.10, T31.11, T31.20, T31.21, T31.22, T31.30, T31.31, T31.32, T31.33, T31.40, T31.41, T31.42, T31.43, T31.44, T31.50, T31.51, T31.52, T31.53, T31.54, T31.55, T31.60, T31.61, T31.62, T31.63, T31.64, T31.65, T31.66, T31.70, T31.71, T31.72, T31.73, T31.74, T31.75, T31.76, T31.77, T31.80, T31.81, T31.82, T31.83, T31.84, T31.85, T31.86, T31.87, T31.88, T31.90, T31.91, T31.92, T31.93, T31.94, T31.95, T31.96, T31.97, T31.98, T31.99<br>Partial thickness (2nd degree) corrosions by size:<br>T32.10, T32.11, T32.20, T32.21, T32.22, T32.30, T32.31, T32.32, T32.33, T32.40, T32.41, T32.42, T32.43, T32.44, T32.50, T32.51, T32.52, T32.53, T32.54, T32.55, T32.60, T32.61, T32.62, T32.63, T32.64, T32.65, T32.66, T32.70, T32.71, T32.72, T32.73, T32.74, T32.75, T32.76, T32.77, T32.80, T32.81, T32.82, T32.83, T32.84, T32.85, T32.86, T32.87, T32.88, T32.90, T32.91, T32.92, T32.93, T32.94, T32.95, T32.96, T32.97, T32.98, T32.99<br><br>**Full thickness burns**<br>Full thickness (3rd degree) injury by anatomic site:<br>T20.3, T20.7, T21.3, T21.7, T22.3, T22.7, T23.3, T23.7, T24.3, T24.7, T25.3, T25.7, T29.3, T29.7, T30.3, T30.7<br>Full thickness (3rd degree) burn injury by size:<br>T31.01, T31.11, T31.12, T31.21, T31.22, T31.23, T31.31, T31.32, T31.33, T31.34, T31.41, T31.42, T31.43, T31.44, T31.45, T31.51, T31.52, T31.53, T31.54, T31.55, T31.56, T31.61, T31.62, T31.63, T31.64, T31.65, T31.66, T31.67, T31.71, T31.72, T31.73, T31.74, T31.75, T31.76, T31.77, T31.78, T31.81, T31.82, T31.83, T31.84, T31.85, T31.86, T31.87, T31.88, T31.89, T31.91, T31.92, T31.93, T31.94, T31.95, T31.96, T31.97, T31.98, T31.99<br>Full thickness (3rd degree) corrosion by size:<br>T32.01, T32.11, T32.12, T32.21, T32.22, T32.23, T32.31, T32.32, T32.33, T32.34, T32.41, T32.42, T32.43, T32.44, T32.45, T32.51, T32.52, T32.53, T32.54, T32.55, T32.56, T32.61, T32.62, T32.63, T32.64, T32.65, T32.66, T32.67, T32.71, T32.72, T32.73, T32.74, T32.75, T32.76, T32.77, T32.78, T32.81, T32.82, T32.83, T32.84, T32.85, T32.86, T32.87, T32.88, T32.89, T32.91, T32.92, T32.93, T32.94, T32.95, T32.96, T32.97, T32.98, T32.99
**Main exposure**	Burn centre referral criteria	Categorical and binary	**Burn injury to special anatomic areas**<br>Face (~ Head and neck): T20.0-T20.7 (except T201, T205), S00.0, S00.2, S00.3, S00.4, S00.5, S00.7, S00.8, S00.9, S01.0, S01.1, S01.2, S01.3, S01.4, S01.5, S01.7, S01.8, S01.9,S09.8<br>Shoulder: S40.7, S40.8, S40.9, S41.0, S41.7, S49.7, S49.8<br>Elbow: S51.0<br>Hip: S70.7, S70.8, S70.9, S71.0, S71.7, S79.7, S79.8<br>Knee: S81.0<br>Hands (~wrist and hand): T23.0-T23.7 (except T231, T235), S60.7, S60.8, S60.9, S61.X, S69.7, S69.8

© 2026 Gus E et al. *JAMA Network Open*.

			Feet: T25.0-T25.7 (except T251, T255), S90.7, S90.8, S90.9, S91.0, S91.1, S91.2, S91.3, S91.7, S99.7, S99.8<br>Perineum: S30.7, S30.8, S30.9, S31.0, S31.2, S31.3, S31.4, S31.5, S31.7, S31.8, S39.7, S39.8<br>Note: Exclude if no T codes in any of the dx10codes1-25<br><br>**Inhalation injury**<br>T27.0-T27.7<br><br>**Chemical burns**<br>~Corrosion<br>T20.4-T20.7; T21.4-T21.7; T22.4-T22.7; T23.4-23.7; T24.4-24.7; T25.4-T25.7; T29.4-T29.7; T30.4-T30.7; T32.0-32.9<br><br>**Electrical burns**<br>W85-W87 Electrical injury<br>From DAD/NACRS/SDS
**Outcome**	Treatment setting for definitive burn care	Categorical and binary	Treatment at a burn centre/non-burn centre<br><br>**Definition of treatment at a burn centre:** one or more encounter(s) at a burn centre within 6 weeks of the index event (=burn injury).<br><br>**Definition of treatment at a non-burn centre:** absence of health records demonstrating receipt of care at a burn centre within 6 weeks of index event.
**Covariates**	Age	Numeric	Age in years, at time of burn episode<br>From RPDB
	Age group	Categorical	Age groups:<br>• 0 - <1 years<br>• 1 – 4 years<br>• 5 – 12 years<br>• 13 – 17 years<br>From RPBD
	Sex	Categorical and binary	Male or female<br>From RPDB
	Material resources	Categorical	Six levels: Q1 (least deprived) to Q5 (most deprived) and missing<br>From ON-MARG
	Children of adolescent mothers	Categorical	Definition: maternal age at birth <20 years old (three levels: children of adolescent mother, children of non-adolescent mother, missing)<br>From MOMBABY
	Immigration status	Categorical	Three levels: refugees/non-refugees/non-immigrant (=no IRCC record)<br>From IRCC
	Rurality	Categorical and binary	Obtained using rural flag (yes/no)<br>From RPBD/PCCF
	Health region with/without burn centres	Categorical and binary	Definition: Residence in LHIN with/without a burn centre.<br>From RPBD (%LHIN macro)
	Time	Numeric	Fiscal year (April 1st to March 31st) 2003-2023

© 2026 Gus E et al. *JAMA Network Open*.

eTable 3. Annual population denominators for Ontario on April 1st of fiscal years 2003-2023

**Population 0-17 years old**	**2003**	**2004**	**2005**	**2006**	**2007**	**2008**	**2009**	**2010**	**2011**	**2012**
	2,991,192	2,989,422	2,984,980	2,968,355	2,952,425	2,941,653	2,924,845	2,914,272	2,909,306	2,904,375
	**2013**	**2014**	**2015**	**2016**	**2017**	**2018**	**2019**	**2020**	**2021**	**2022**
	2,891,317	2,881,244	2,874,820	2,882,251	2,896,291	2,908,120	2,929,635	2,935,369	2,941,475	2,950,790
**Age in years**	**2003**	**2004**	**2005**	**2006**	**2007**	**2008**	**2009**	**2010**	**2011**	**2012**
0 - <1 years old	270,312	274,742	275,878	279,441	284,950	287,673	277,433	275,449	283,891	284,557
1 – 4 years old	560,252	554,653	553,191	557,243	558,268	565,757	574,200	581,270	579,133	580,675
5 – 12 years old	1,318,475	1,300,820	1,281,458	1,259,701	1,241,940	1,220,725	1,205,396	1,196,316	1,193,390	1,196,942
13 – 17 years old	842,153	859,207	874,453	871,970	867,267	867,498	867,816	861,237	852,892	842,201
	**2013**	**2014**	**2015**	**2016**	**2017**	**2018**	**2019**	**2020**	**2021**	**2022**
0 - <1 years old	282,153	280,226	280,083	280,348	280,446	279,632	279,186	276,133	276,325	276,138
1 – 4 years old	578,325	576,770	581,866	583,199	584,781	583,985	586,825	589,466	583,403	576,432
5 – 12 years old	1,203,840	1,215,424	1,215,406	1,229,620	1,242,682	1,255,274	1,267,079	1,273,269	1,271,325	1,272,999
13 – 17 years old	826,999	808,824	797,465	789,084	788,382	789,229	796,545	796,501	810,422	825,221
**Sex**	**2003**	**2004**	**2005**	**2006**	**2007**	**2008**	**2009**	**2010**	**2011**	**2012**
Male	1,534,328	1,533,037	1,530,635	1,522,277	1,513,843	1,508,381	1,499,386	1,494,310	1,491,436	1,488,869
Female	1,456,864	1,456,385	1,454,345	1,446,078	1,438,582	1,433,272	1,425,459	1,419,962	1,417,870	1,415,506
	**2013**	**2014**	**2015**	**2016**	**2017**	**2018**	**2019**	**2020**	**2021**	**2022**
Male	1,481,541	1,476,293	1,472,983	1,477,086	1,484,527	1,490,286	1,500,671	1,503,936	1,507,225	1,511,797
Female	1,409,776	1,404,951	1,401,837	1,405,165	1,411,764	1,417,834	1,428,964	1,431,433	1,434,250	1,438,993
**Material resources**	**2003**	**2004**	**2005**	**2006**	**2007**	**2008**	**2009**	**2010**	**2011**	**2012**
Quintile 1 (least deprived)	649,962	608,761	635,706	659,206	679,996	694,027	533,783	527,867	522,886	519,603
Quintile 2	581,293	584,921	587,247	586,001	584,219	581,683	567,644	567,998	568,756	569,049
Quintile 3	552,529	561,272	556,049	548,883	543,370	539,539	556,369	557,772	560,083	562,699
Quintile 4	538,596	546,158	535,468	523,167	511,281	502,922	562,603	562,607	563,594	563,467
Quintile 5 (most deprived)	589,117	643,290	626,296	607,271	590,436	580,637	677,716	671,596	667,865	663,580
Missing	79,695	45,020	44,214	43,827	43,123	42,845	26,730	26,432	26,122	25,977
	**2013**	**2014**	**2015**	**2016**	**2017**	**2018**	**2019**	**2020**	**2021**	**2022**
Quintile 1 (least deprived)	517,938	634,879	645,888	658,919	671,710	682,393	577,761	582,791	591,265	595,885
Quintile 2	570,565	605,543	607,429	610,483	614,684	618,734	652,634	659,739	667,706	673,696
Quintile 3	561,606	535,518	532,106	531,230	530,400	530,332	597,584	597,834	598,628	599,646
Quintile 4	561,422	501,197	494,494	491,420	490,696	489,784	501,616	500,970	499,104	499,165
Quintile 5 (most deprived)	653,732	570,217	561,397	556,672	554,397	551,644	570,190	564,621	555,165	552,633
Missing	26,054	33,890	33,506	33,527	34,404	35,233	29,850	29,414	29,607	29,765

© 2026 Gus E et al. *JAMA Network Open*.

eTable 3 continued

Children of adolescent mothers	2003	2004	2005	2006	2007	2008	2009	2010	2011	2012
Yes	77,933	82,104	86,259	90,186	92,156	93,852	94,855	96,477	95,280	92,436
No	1,672,018	1,788,574	1,905,003	2,013,513	2,049,861	2,080,689	2,113,207	2,182,167	2,213,362	2,221,920
No mother-baby linkage	1,241,241	1,118,744	993,718	864,656	810,408	767,112	716,783	635,628	600,664	590,019
	**2013**	**2014**	**2015**	**2016**	**2017**	**2018**	**2019**	**2020**	**2021**	**2022**
Yes	88,518	84,954	81,523	78,067	74,484	71,085	67,666	63,828	60,356	55,693
No	2,224,452	2,231,241	2,240,667	2,251,479	2,264,713	2,278,162	2,293,126	2,290,416	2,301,295	2,178,526
No mother-baby linkage	578,347	565,049	552,630	552,705	557,094	558,873	568,843	581,125	579,824	716,571
**Residence type**	**2003**	**2004**	**2005**	**2006**	**2007**	**2008**	**2009**	**2010**	**2011**	**2012**
Rural	388,072	363,089	355,985	347,691	340,995	335,254	327,372	322,381	316,966	312,545
Urban	2,600,718	2,624,574	2,627,410	2,619,374	2,610,328	2,605,299	2,596,018	2,590,408	2,590,795	2,590,336
Missing	2,402	1,759	1,585	1,290	1,102	1,100	1,455	1,483	1,545	1,494
	**2013**	**2014**	**2015**	**2016**	**2017**	**2018**	**2019**	**2020**	**2021**	**2022**
Rural	307,602	283,239	281,410	281,596	283,109	284,401	278,998	280,166	286,015	289,767
Urban	2,582,177	2,591,193	2,586,837	2,593,840	2,605,643	2,615,441	2,645,077	2,650,058	2,650,332	2,655,887
Missing	1,538	6,812	6,573	6,815	7,539	8,278	5,560	5,145	5,128	5,136
**Immigration status**	**2003**	**2004**	**2005**	**2006**	**2007**	**2008**	**2009**	**2010**	**2011**	**2012**
Canadian born	2,768,957	2,765,320	2,760,080	2,741,206	2,727,985	2,721,651	2,707,585	2,700,271	2,695,085	2,694,121
Non-refugee immigrants	183,960	185,800	186,400	189,350	187,888	185,055	183,605	180,532	180,812	176,578
Refugee immigrants	38,275	38,302	38,500	37,799	36,552	34,947	33,655	33,469	33,409	33,676
	**2013**	**2014**	**2015**	**2016**	**2017**	**2018**	**2019**	**2020**	**2021**	**2022**
Canadian born	2,686,746	2,681,674	2,684,116	2,692,206	2,707,505	2,723,436	2,746,997	2,753,524	2,775,098	2,803,350
Non-refugee immigrants	171,702	167,341	158,826	153,225	147,679	140,406	136,142	133,396	121,820	107,361
Refugee immigrants	32,869	32,229	31,878	36,820	41,107	44,278	46,496	48,449	44,557	40,079

© 2026 Gus E et al. *JAMA Network Open*.

eTable 4. Likelihood of being treated at a burn centre for pediatric burn injuries with and without burn centre referral criteria in Ontario, Canada, 2003 to 2023 (with parameters estimates for covariates)

Parameter	Burn episodes treated at burn centres<br>n / N (%)	Model 1[1] Time n=79,782<br>aRR (95% CI)	Model 2[2] Time and demographic characteristics n=79,782<br>aRR (95% CI)	Model 3[3] Time, demographic, social vulnerability and care accessibility factors n=79,782<br>aRR (95% CI)
**Overall burn episodes**	13,531 / 79,782 (17)	-	-	-
**Burn centre referral criteria**				
No burn centre referral criterion*	5,998 / 44,970 (13)	1.00 (Reference)	1.00 (Reference)	1.00 (Reference)
Any burn centre referral criteria	7,533 / 34,812 (22)	1.61 (1.56 – 1.66)	1.53 (1.48 – 1.58)	1.50 (1.46 – 1.54)
**Fiscal year**	-	1.06 (1.06 – 1.07)	1.06 (1.06 – 1.06)	1.06 (1.06 – 1.06)
**Age (years)**				
0 - <1	2,057 / 8,015 (26)		2.49 (2.35 – 2.63)	2.35 (2.23 – 2.48)
1 – 4	6,675 / 32,853 (20)		1.97 (1.87 – 2.06)	1.87 (1.79 – 1.96)
5 – 12	2,830 / 18,143 (16)		1.59 (1.51 – 1.68	1.52 (1.44 – 1.60)
13 – 17*	1,969 / 20,771 (9.5)		1.00 (Reference)	1.00 (Reference)
**Sex**				
Male	7,644 / 44,191 (17)		1.00 (0.97 – 1.03)	1.02 (0.99 – 1.05)
Female*	5,887 / 35,591 (17)		1.00 (Reference)	1.00 (Reference)
**Material resources quintile**				
Quintile 1 (least deprived)*	2,920 / 13,483 (22)			1.00 (Reference)
Quintile 2	2,249 / 14,933 (15)			0.88 (0.84 – 0.92)
Quintile 3	2,077 / 15,260 (14)			0.86 (0.82 – 0.90)
Quintile 4	2,341 / 15,498 (15)			0.93 (0.89 – 0.98)
Quintile 5 (most deprived)	3,765 / 19,067 (20)			0.99 (0.95 – 1.03)
Missing	179 / 1,541 (12)			1.17 (1.00 – 1.37)
**Children of adolescent mothers**				
Yes	533 / 3,845 (14)			0.91 (0.84 – 0.97)
No*	10,660 / 60,712 (18)			1.00 (Reference)
Missing	2,338 / 15,225 (15)			1.07 (1.03 – 1.11)
**Rurality**				
Rural residence	765 / 16,164 (4.7)			0.26 (0.24 – 0.27)
Urban residence*	12,725 / 63,513 (20)			1.00 (Reference)
Missing	41 / 105 (39)			1.22 (0.94 – 1.57)
**Health Region with/without burn centres**				
Health region with burn centre*	9,754 / 30,000 (33)			1.00 (Reference)
Health region without burn centre	3,777 / 49,782 (7.6)			0.23 (0.22 – 0.24)

© 2026 Gus E et al. *JAMA Network Open*.

eTable 4 continued

Parameter	Burn episodes treated at burn centres n / N (%)	Model 1[1] n=79,782 aRR (95% CI)	Model 2[2] n=79,782 aRR (95% CI)	Model 3[3] n=79,782 aRR (95% CI)
**Immigrant status**				
Non-immigrants*	13,064 / 77,444 (17)			1.00 (Reference)
Non-refugee immigrants	257 / 1,636 (16)			1.19 (1.07 – 1.33)
Refugee immigrants	210 / 702 (30)			1.51 (1.36 – 1.68)

Legend
[1] Model 1 = adjusted for fiscal year
[2] Model 2 = adjusted for fiscal year, age and sex
[3] Model 3 = adjusted for fiscal year, age, sex, material resources quintiles, children of adolescent mothers, rural residence, health region with/without burn centres, and immigration status
TBSA = total body surface area; aRR = adjusted rate ratio; CI = confidence interval; * = reference; n=burn episodes treated at burn centres; N=all burn episodes

© 2026 Gus E et al. *JAMA Network Open*.

eTable 5. Likelihood of being treated at a burn centre for pediatric burn injuries with one or more burn centre referral criteria in Ontario, Canada, 2003 to 2023 (with parameters estimates for covariates)

	Burn episodes treated at burn centres	Model 1[1] Time n=79,782	Model 2[2] Time and demographic characteristics n=79,782	Model 3[3] Time, demographic, social vulnerability and care accessibility factors n=79,782
**Parameter**	**n / N (%)**	**aRR (95% CI)**	**aRR (95% CI)**	**aRR (95% CI)**
**Overall burn episodes**	13,531 / 79,782 (17)	-	-	-
**Burn centre referral criteria**				
No burn centre referral criterion*	5,998 / 44,970 (13)	1.00 (Reference)	1.00 (Reference)	1.00 (Reference)
1 burn centre referral criterion	5,398 / 29,768 (18)	1.36 (1.31 – 1.40)	1.30 (1.25 – 1.34)	1.27 (1.23 – 1.30)
2 burn centre referral criteria	1,748 / 4,459 (39)	2.83 (2.71 – 2.95)	2.62 (2.51 – 2.73)	2.63 (2.51 – 2.75)
3+ burn centre referral criteria	387 / 585 (66)	4.72 (4.44 – 5.03)	4.40 (4.12 – 4.70)	4.71 (4.32 – 5.15)
**Fiscal year**	-	1.06 (1.06 – 1.06)	1.06 (1.06 – 1.06)	1.06 (1.05 – 1.06)
**Age (years)**				
0 - <1	2,057 / 8,015 (26)		2.41 (2.28 – 2.54)	2.27 (2.15 – 2.40)
1 – 4	6,675 / 32,853 (20)		1.95 (1.86 – 2.04)	1.86 (1.78 – 1.94)
5 – 12	2,830 / 18,143 (16)		1.59 (1.51 – 1.68)	1.53 (1.45 – 1.60)
13 – 17*	1,969 / 20,771 (9.5)		1.00 (Reference)	1.00 (Reference)
**Sex**				
Male	7,644 / 44,191 (17)		1.00 (0.97 – 1.03)	1.02 (0.99 – 1.05)
Female*	5,887 / 35,591 (17)		1.00 (Reference)	1.00 (Reference)
**Material resources quintile**				
Quintile 1 (least deprived)*	2,920 / 13,483 (22)			1.00 (Reference)
Quintile 2	2,249 / 14,933 (15)			0.87 (0.83 – 0.91)
Quintile 3	2,077 / 15,260 (14)			0.85 (0.81 – 0.89)
Quintile 4	2,341 / 15,498 (15)			0.91 (0.87 – 0.95)
Quintile 5 (most deprived)	3,765 / 19,067 (20)			0.97 (0.93 – 1.01)
Missing	179 / 1,541 (12)			1.17 (1.00 – 1.37)
**Children of adolescent mothers**				
Yes	533 / 3,845 (14)			0.89 (0.83 – 0.96)
No*	10,660 / 60,712 (18)			1.00 (Reference)
Missing	2,338 / 15,225 (15)			1.06 (1.02 – 1.11)
**Rurality**				
Rural residence	765 / 16,164 (4.7)			0.26 (0.24 – 0.28)
Urban residence*	12,725 / 63,513 (20)			1.00 (Reference)
Missing	41 / 105 (39)			1.24 (0.96 – 1.60)

© 2026 Gus E et al. *JAMA Network Open*.

eTable 5 continued

	Burn episodes treated at burn centres	Model 1[1] n=79,782	Model 2[2] n=79,782	Model 3[3] n=79,782
**Parameter**	**n / N (%)**	**aRR (95% CI)**	**aRR (95% CI)**	**aRR (95% CI)**
**Health region with/without burn centres**				
Health region with burn centre*	9,754 / 30,000 (33)			1.00 (Reference)
Health region without burn centre	3,777 / 49,782 (7.6)			0.23 (0.22 – 0.24)
**Immigrant status**				
Non-immigrants*	13,064 / 77,444 (17)			1.00 (Reference)
Non-refugee immigrants	257 / 1,636 (16)			1.20 (1.08 – 1.34)
Refugee immigrants	210 / 702 (30)			1.56 (1.41 – 1.73)

Legend
[1] Model 1 = adjusted for fiscal year
[2] Model 2 = adjusted for fiscal year, age and sex
[3] Model 3 = adjusted for fiscal year, age, sex, material resources quintiles, children of adolescent mothers, rural residence, health region with/without burn centres, and immigration status
TBSA = total body surface area; aRR = adjusted rate ratio; CI = confidence interval; * = reference; n=burn episodes treated at burn centres; N=all burn episodes

© 2026 Gus E et al. *JAMA Network Open*.

eTable 6. Likelihood of being treated at a burn centre for pediatric burn injuries with mutually exclusive specific burn centre referral criteria in Ontario, Canada, 2003 to 2023 (with parameters estimates for covariates)

	Burn episodes treated at burn centres	Model 1[1] Time n=79,782	Model 2[2] Time and demographic characteristics n=79,782	Model 3[3] Time, demographic, social vulnerability and care accessibility factors n=79,782
**Parameter**	**n / N (%)**	**aRR (95% CI)**	**aRR (95% CI)**	**aRR (95% CI)**
**Overall burn episodes**	13,531 / 79,782 (17)	-	-	-
**Burn centre referral criteria**				
No burn centre referral criterion*	5,998 / 44,970 (13)	1.00 (Reference)	1.00 (Reference)	1.00 (Reference)
>10% TBSA partial thickness burn	247 / 1,097 (23)	1.65 (1.48 – 1.85)	1.52 (1.36 – 1.70)	1.66 (1.49 – 1.84)
Full-thickness burn	555 / 2,286 (24)	1.72 (1.59 – 1.85)	1.68 (1.56 – 1.81)	1.66 (1.54 – 1.78)
Burns to special anatomic areas	4,012 / 23,030 (17)	1.32 (1.27 – 1.37)	1.25 (1.20 – 1.29)	1.21 (1.17 – 1.25)
Inhalation injury	14 / 56 (25)	1.86 (1.20 – 2.89)	2.15 (1.40 – 3.29)	1.88 (1.21 – 2.91)
Chemical injury	318 / 1,192 (27)	1.93 (1.75 – 2.12)	1.83 (1.67 – 2.01)	1.80 (1.65 – 1.97)
Electrical injury	252 / 2,107 (12)	0.88 (0.78 – 0.99)	0.91 (0.81 – 1.02)	0.93 (0.84 – 1.04)
2+ burn centre referral criteria	2,135 / 5,044 (42)	3.05 (2.93 – 3.18)	2.83 (2.72 – 2.95)	2.86 (2.75 – 2.98)
**Fiscal year**	-	1.06 (1.06 - 1.06)	1.06 (1.06 – 1.06)	1.05 (1.05 – 1.06)
**Age (years)**				
0 - <1	2,057 / 8,015 (26)		2.37 (2.25 – 2.51)	2.23 (2.11 – 2.35)
1 – 4	6,675 / 32,853 (20)		1.95 (1.86 -2.05)	1.86 (1.77 – 1.94)
5 – 12	2,830 / 18,143 (16)		1.59 (1.51 – 1.68)	1.52 (1.45 – 1.60)
13 – 17*	1,969 / 20,771 (9.5)		1.00 (Reference)	1.00 (Reference)
**Sex**				
Male	7,644 / 44,191 (17)		1.00 (0.97 – 1.03)	1.02 (1.00 – 1.05)
Female*	5,887 / 35,591 (17)		1.00 (Reference)	1.00 (Reference)
**Material resources quintile**				
Quintile 1 (least deprived)*	2,920 / 13,483 (22)			1.00 (Reference)
Quintile 2	2,249 / 14,933 (15)			0.86 (0.83 – 0.90)
Quintile 3	2,077 / 15,260 (14)			0.84 (0.81 – 0.89)
Quintile 4	2,341 / 15,498 (15)			0.91 (0.87 – 0.95)
Quintile 5 (most deprived)	3,765 / 19,067 (20)			0.96 (0.92 – 1.00)
Missing	179 / 1,541 (12)			1.17 (1.00 – 1.37)
**Children of adolescent mothers**				
Yes	533 / 3,845 (14)			0.89 (0.83 – 0.96)
No*	10,660 / 60,712 (18)			1.00 (Reference)
Missing	2,338 / 15,225 (15)			1.06 (1.02 – 1.11)

© 2026 Gus E et al. *JAMA Network Open*.

eTable 6 continued

Parameter	Burn episodes treated at burn centres n / N (%)	Model 1[1] n=79,782 aRR (95% CI)	Model 2[2] n=79,782 aRR (95% CI)	Model 3[3] n=79,782 aRR (95% CI)
**Rurality**				
Rural residence	765 / 16,164 (4.7)			0.26 (0.24 – 0.28)
Urban residence*	12,725 / 63,513 (20)			1.00 (Reference)
Missing	41 / 105 (39)			1.21 (0.93 – 1.56)
**Health regions with/without burn centres**				
HCCSS with burn centre*	9,754 / 30,000 (33)			1.00 (Reference)
HCCSS without burn centre	3,777 / 49,782 (7.6)			0.23 (0.22 – 0.24)
**Immigrant status**				
Non-immigrants*	13,064 / 77,444 (17)			1.00 (Reference)
Non-refugee immigrants	257 / 1,636 (16)			1.19 (1.07 – 1.32)
Refugee immigrants	210 / 702 (30)			1.53 (1.38 – 1.70)

Legend
[1] Model 1 = adjusted for fiscal year
[2] Model 2 = adjusted for fiscal year, age and sex
[3] Model 3 = adjusted for fiscal year, age, sex, material resources quintiles, children of adolescent mothers, rural residence, health regions with/without burn centres, and immigration status
TBSA = total body surface area; aRR = adjusted rate ratio; CI = confidence interval; * = reference; n=burn episodes treated at burn centres; N=all burn episodes

© 2026 Gus E et al. *JAMA Network Open*.
