# Supplementary material for: Adherence to Burn Center Referral Criteria for Pediatric Burns
Source: JAMA Netw Open. 2026 Feb 12;9(2):e2559159. doi: 10.1001/jamanetworkopen.2025.59159 (PMC12902878; doi:10.1001/jamanetworkopen.2025.59159)
Supplement: Supplement 2. — Data Sharing Statement [file jamanetwopen-e2559159-s002.pdf]

Data Sharing Statement

Gus. Adherence to Burn Center Referral Criteria for Pediatric Burns. *JAMA Netw Open*. Published online February 11, 2026. doi:10.1001/jamanetworkopen.2025.59159

Data
Data available: No

Additional Information
Explanation for why data not available: The data sets from this study are held securely in coded form at ICES. Data-sharing agreements prohibit ICES from making the data sets publicly available, but access may be granted to those who meet pre-specified criteria for confidential access, available at www.ices.on.ca/DAS. The complete data set creation plan, and underlying analytic code are available from the authors upon request, understanding that the programs may rely upon coding templates or macros unique to ICES.
